# Cost-minimization analysis of immunoglobulin treatment of primary immunodeficiency diseases in Spain

**DOI:** 10.1007/s10198-021-01378-x

**Published:** 2021-09-21

**Authors:** Laia Alsina, J. Bruno Montoro, Pedro Moral Moral, Olaf Neth, Marta Ortiz Pica, Silvia Sánchez-Ramón, María Presa, Itziar Oyagüez, Miguel Ángel Casado, Luis Ignacio González-Granado

**Affiliations:** 1 Clinical Immunology and Primary Immunodeficiencies Unit, Pediatric Allergy and Clinical Immunology Department, Institut de Recerca Sant Joan de Déu, Hospital Sant Joan de Déu, Universitat de Barcelona, Barcelona, Spain; 2 Pharmacy Service, Hospital Universitari Vall d’Hebron, Barcelona, Spain; 3 Sección de Inmunopatología Y Enfermedades Minoritarias, Hospital Universitari I Politècnic La Fe, Valencia, Spain; 4 Paediatric Infectious Diseases, Rheumatology and Immunology Unit, Hospital Universitario Virgen del Rocío/Instituto de Biomedicina de Sevilla (IBiS), Sevilla, Spain; 5 Hospital de Día Médico, Hospital Clínico San Carlos, Madrid, Spain; 6 Departamento de Inmunología Clínica, IML, Hospital Clínico San Carlos, Universidad Complutense of Madrid, Madrid, Spain; 7 Pharmacoeconomics and Outcomes Research Iberia (PORIB), Madrid, Spain; 8 Primary Immunodeficiencies Unit, Pediatrics, University Hospital 12 Octubre/Research Institute Hospital, 12 octubre (i+12), Madrid, Spain; 9 Pediatrics, School of Medicine, Complutense University, Madrid, Spain

**Keywords:** Primary immunodeficiency disease, Immune system, Immunoglobulin replacement therapy, Subcutaneous immunoglobulin, Intravenous immunoglobulin, Cost-minimization analysis, I11

## Abstract

Primary immunodeficiency diseases (PID), which are comprised of over 400 genetic disorders, occur when a component of the immune system is diminished or dysfunctional. Patients with PID who require immunoglobulin (IG) replacement therapy receive intravenous IG (IVIG) or subcutaneous IG (SCIG), each of which provides equivalent efficacy. We developed a cost-minimization model to evaluate costs of IVIG versus SCIG from the Spanish National Healthcare System perspective. The base case modeled the annual cost per patient of IVIG and SCIG for the mean doses (per current expert clinical practice) over 1 year in terms of direct (drug and administration) and indirect (lost productivity for adults and parents/guardians of pediatric patients) costs. It was assumed that all IVIG infusions were administered in a day hospital, and 95% of SCIG infusions were administered at home. Drug costs were calculated from ex-factory prices obtained from local databases minus the mandatory deduction. Costs were valued on 2018 euros. The annual modeled costs were €4,266 lower for patients with PID who received SCIG (total €14,466) compared with those who received IVIG (total €18,732). The two largest contributors were differences in annual IG costs as a function of dosage (– €1,927) and hospital administration costs (– €2,688). However, SCIG incurred training costs for home administration (€695). Sensitivity analyses for two dose-rounding scenarios were consistent with the base case. Our model suggests that SCIG may be a cost-saving alternative to IVIG for patients with PID in Spain.

**Supplementary Information:**

The online version contains supplementary material available at 10.1007/s10198-021-01378-x.

## Introduction

Primary immunodeficiency diseases (PID) occur when a component of the immune system is diminished or dysfunctional and may be caused by over 400 identified genetic disorders [1]. PID may result in frequent or serious infections, autoimmune disorders, systemic inflammation, and/or cancer, all of which can lead to significant morbidity and mortality [2, 3]. The prevalence of PID in Spain is estimated to be at least 4.9 per 100,000 individuals [4]. However, because this calculation is based on registry data, the actual prevalence is likely to be higher.

Typically, patients who have PID that is associated with defects in antibody production receive immunoglobulin-replacement therapy (IGRT). Various IGRT products are available in subcutaneous immunoglobulin (SCIG), facilitated SCIG (fSCIG) and intravenous immunoglobulin (IVIG) formulations in Spain. Typically, IVIG is administered by a healthcare professional in a hospital outpatient clinic once every 3–4 weeks, and SCIG is administered at home once every 1–4 weeks. IVIG and SCIG formulations offer similar levels of efficacy [5, 6], but SCIG produces fewer systemic adverse events [5, 7, 8] than does IVIG. SCIG provides the patient with the convenience to self-infuse at home, whereas IVIG does not. Facilitated SCIG treatment has two components: IgG 10% and recombinant human hyaluronidase (rHuPh20). rHuPH20 is infused first resulting in a transient and local increase in subcutaneous tissue permeability, allowing larger doses of immunoglobulin (IG) to be administered every 3–4 weeks [9–13].

Healthcare professionals and patients may consider these aspects of IVIG and SCIG treatments differently, depending on the patient’s conditions, preferences, and perceived treatment burdens. With the understanding that patient preference plays a large role in the choice of IG therapy, we sought to understand the cost implications of patients selecting home-based SCIG or hospital-based IVIG in the Spanish healthcare setting. This study calculated and compared the annual cost of IVIG and SCIG as part of the pharmaceutical services delivered by the Spanish National Healthcare System (SNS) for the treatment of PID.

## Methods

### General overview

**Fig. 1:**
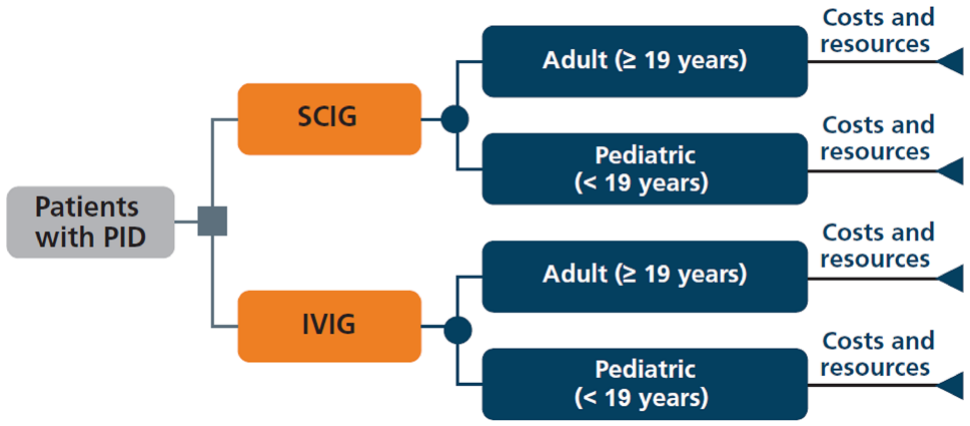
Structure of the cost-minimization analysis model. *IVIG* intravenous immunoglobulin, *PID* primary immunodeficiency diseases, *SCIG* subcutaneous immunoglobulin

A cost-minimization analysis was developed based on the decision tree shown in Fig. 1. The model included both direct (i.e., IG therapy, premedication, hospital administration, home training, dispensing) and indirect (i.e., work absenteeism) costs. The analysis was from the SNS and societal perspectives, with a time horizon of 1 year.

Because the premise of a cost-minimization analysis assumes that the therapies being compared have equivalent outcomes, a literature review was conducted to establish the therapeutic equivalence of SCIG and IVIG. Results of two studies, one being a noninferiority trial [6] and the other a meta-analysis of 47 clinical studies [5], found no differences in efficacy between SCIG and IVIG, as measured by serum IG levels and infection rates [5, 6]. Another meta-analysis of 24 observational studies also found no significant difference in overall infections or serious infections for SCIG and IVIG, although a statistically significant association between higher IG trough levels and lower infection rates was observed with SCIG but not IVIG [14]. We took a conservative approach and assumed equivalent efficacy of SCIG and IVIG for this analysis.

### Population assumptions

In the model, patients receiving SCIG could receive either a conventional 20% concentration SCIG product or a 10% concentration facilitated SCIG product, and those receiving IVIG could receive either a 5 or 10% concentration product. The usage ratios of IVIG and SCIG and each treatment available in Spain in every category were determined by current expert clinical practice and are described in Online Resource 1. The ratio of 52.5% adult (≥ 19 years) and 47.5% pediatric (< 19 years) cases was based on European Society for Immunodeficiencies database estimates for Europe [4]. This ratio was applied to patients receiving IVIG and patients receiving SCIG. More detailed age-distribution assumptions are shown in Online Resource 2.

Dosing for IG therapy is based on the patient’s body weight (g/kg); therefore, the mean weight of adult and pediatric patients was included in the model to calculate IG doses. Mean weight for adults was assumed to be 70 kg, based on Spanish Hospital Pharmacy Society (SEFH) guidelines for economic evaluations [15]. For pediatric patients, mean weight was categorized into four age groups and calculated based on data published by the Instituto de Investigación sobre Crecimiento y Desarrollo [16]. Mean weights by age group were: < 5 years, 12.38 kg; 5–9 years, 25.88 kg; 10–15 years, 47.04 kg; and 16–18 years, 62.16 kg.

Employment-status and education-level estimates were used in the calculation of social resources (e.g., work absenteeism, school absenteeism, and lost leisure time) that were consumed by the time it takes to administer IGRT. All (100%) pediatric patients were assumed to be attending school. The overall employment rate of the Spanish population is 63.74% [17]. Clinical experts from the Spanish Association of Patients with Primary Immunodeficiencies suggested approximately 70% of patients who have PID and are of working age are employed. Therefore, we multiplied the overall Spanish employment rate by 70% to calculate an estimated employment rate of 44.6% for patients aged ≥ 19 and ≤ 64 years in our study population. Parents/guardians of pediatric patients, who often must travel with their children for treatment at the hospital, were assumed to be employed at similar rates as the Spanish general population [17].

### Model inputs

Prescribing information for each IGRT product provides a range, or interval, for dosing. Therefore, mean doses were based on current expert clinical practice and clinical guidelines [18–20]. The model assumed that patients were treatment naïve, and the recommended starting and maintenance doses of IGRT based on each product’s prescribing information were used [10, 21, 22]. In Europe, the monthly dosage ratio of IVIG to SCIG is 1:1 [23]. Due to differences in the prescribing information for conventional and facilitated SCIG, dosage and dosing frequencies were calculated separately. Dosages and dosing frequencies, also determined by current expert clinical practice, are shown in Table 1.

**Table 1:** Facilitated SCIG, conventional SCIG, and IVIG dosage in PID

	SCIG						IVIG		
	Facilitated			Conventional					
	Percentage of patients	Dose (g/kg)	Frequency	Percentage of patients	Dose (g/kg)	Frequency	Percentage of patients	Dose (g/kg)	Frequency
Starting dose									
Adult	–	0.40	Every 3 weeks for 3 months	–	0.10	Every 24 h for 5 days	–	0.40	Every 3 weeks for 3 months
Pediatric	–	0.60	Every 4 weeks for 3 months	–	0.10	Every 24 h for 5 days	–	0.60	Every 4 weeks for 3 months
Maintenance dose									
Adult	–	0.40	Every 4 weeks	60	0.20	Every 2 weeks	–	0.40	Every 4 weeks
				40	0.10	Every 7 days			
Pediatric	–	0.50	Every 4 weeks	60	0.20	Every 2 weeks	–	0.60	Every 4 weeks
				40	0.10	Every 7 days			
Adjusted dosage									
Adult	20	0.50	Every 4 weeks	3	0.30	Every 2 weeks	21	0.50	Every 4 weeks
				2	0.20	Every 7 days	4	0.50	Every 3 weeks
Pediatric	20	0.60	Every 4 weeks	3	0.30	Every 2 weeks	21	0.70	Every 4 weeks
				2	0.20	Every 7 days	4	0.70	Every 3 weeks

*IVIG* intravenous immunoglobulin, *PID* primary immunodeficiency diseases, *SCIG* subcutaneous immunoglobulin

All IVIG infusions were assumed to be administered in a day hospital, and most SCIG infusions were assumed to be administered in the patient’s home. On the basis of current expert clinical practice, we assumed that a small percentage of patients (1–5%, depending on the product) taking SCIG would receive their infusions in a day hospital, rather than at home. The cost of the infusion in the day hospital was assumed to include all services provided, including a proportion of healthcare-provider salaries and materials used; however, capital costs were not included. The costs for the day hospital for adults (€175.45 per visit per adult patient) and pediatric patients (€228.64 per visit per pediatric patient) and time for pharmacy dispensing (€29.84) were obtained from the eSalud database of local costs, considering an average of the individual costs available [24]. eSalud is a private database proprietary of Oblikue, available by subscription. It includes data about unitary costs for health resources from different sources in Spain, such as scientific literature, official tariffs from autonomous regions, and costs estimated by the Ministry of Health. For patients receiving SCIG infusions at home, experts agreed that three or four hospital training sessions (3.5 days, 2 h each day) with an experienced professional would be needed at treatment initiation to teach the patient or caregiver how to use the infusion devices and how to recognize and possibly manage adverse reactions. The total cost of the training sessions per patient (adult or pediatric) was estimated to be €694.90 (Online resource 5) based on the estimated cost of nurse consults.

The model did not account for potential differences in adherence between patients receiving SCIG and IVIG; however, such differences are likely to be small based on real-world data [25]. Nor did it allow for potential switching between SCIG and IVIG, since it was assumed that patients were correctly assessed at the start of receiving IGRT, and no patients would switch their route of administration during the 1-year period. The model assumed that all patients-receiving SCIG at home would either be capable of self-administering treatment (adults) or have their caregiver administer treatment (in the case of children).

In addition, the model assumed patients or caregivers visit the hospital pharmacy at least four times (4 days) annually on average to obtain the SCIG medication. This is based on author consensus that patients or caregivers typically collect their medication every 3 months.

Due to the higher rate of systemic reactions associated with IVIG compared with SCIG [5, 7, 8], we assumed based on current expert clinical practice that 15% of patients receiving IVIG would require premedication (e.g., acetaminophen, corticosteroid, antihistamine). The premedication dosages for adult and pediatric patients-receiving IVIG are shown in Online Resource 3. The cost of premedication was obtained from the manufacturer price, after applying the deduction required by Spanish Royal Decree Law (RDL) 8/2010 [26].

Daily-life indicators affected by IGRT included work absenteeism, school absenteeism, and loss of leisure time. Time consumed by IGRT included time to prepare and infuse IG and premedication as well as to travel to the day hospital and from the hospital pharmacy (Online Resource 4).

Work absenteeism for adult (≥ 19 years) patients receiving IVIG was assumed to include time spent in the day hospital and travel time to the hospital. For employed adult patients receiving SCIG, this included training sessions, SCIG infusion in the day hospital (for a small percentage of patients), and travel to the hospital for training or infusions. Preparing, performing, and cleaning up SCIG infusions at home were assumed to have no impact on work absenteeism because the patient can administer SCIG at home outside of work hours. The time spent for these activities also applied to working parents/guardians of pediatric patients. For working patients and parents/guardians of pediatric patients, an average hourly wage (€14.04) was applied to all work absenteeism time, based on National Statistics Institute data [27].

School absenteeism and lost leisure time were calculated to show the impacts of IGRT on patient’s, parent’s, and guardian’s time; however, these data were not included in the indirect cost calculation because there was no associated cost. School absenteeism included the time that pediatric (< 19 years) patients spent on preparation and infusion of IVIG and premedication, preparation and infusion of SCIG administered in a day hospital, training sessions for SCIG, and travel time. For retired or unemployed adult patients, pediatric patients, and unemployed parents/guardians caring for pediatric patients, IG infusions were assumed to affect other activities not related to work and school, including leisure time, which was considered a social loss. Leisure time could be impacted due to administration in a day hospital, training sessions, home infusions, and travel time for infusions, training, and SCIG dispensing.

All unit costs were valued in 2018 euros (€). Costs of IG therapies were determined from the ex-factory price following the application of the deduction established by RDL 8/2010 [26]. All unit costs were obtained from the Bot Plus 2.0 database [28]. Cost inflation was not calculated because the time horizon for the analysis was only 1 year.

### Analyses

The base case considered the cost of SCIG and IVIG per the mean dose established by current expert clinical practice (Table 1) multiplied by the ex-factory price per milligram of each of the IG therapies. The total costs of SCIG and IVIG represent a weighted average of adult and pediatric patients in each group. Two scenario analyses were also conducted to model the impacts of using SEFH guidelines and current expert clinical practice. In both scenarios, IGRT dosage was calculated by vial rather than exact dose in milligrams. In scenario 1, vial adjustment was performed by adjusting to the nearest lower dosage in adult patients and the nearest higher dosage in pediatric patients, per SEFH 2011 guidance to reduce pharmacy cost and conserve drug volumes [29]. For instance, if the total calculated dosage for an adult patient ended in 0.3 g, and the smallest vial for the IG product was 0.5 g, the dose would be rounded down to the nearest whole number of grams to conserve vials. A dose ending in 0.3 g for a pediatric patient for the same product would be rounded up to the nearest 0.5 g. In scenario 2, reflecting real clinical practice, vial adjustment was carried out by rounding up to the nearest higher-dosage unit in both adult and pediatric patients.

## Results

In the base case (Table 2), the annual cost of SCIG treatment per average patient was lower than the IVIG cost by €4266.17 (22.8%). Patients receiving SCIG were estimated to lose fewer hours of work and school time per year as a result of treatment administration and associated travel compared with those receiving IVIG (79.2 vs 101.1 h, respectively). This contributed to lower annual indirect costs, in terms of work productivity for working adult patients and working parents/guardians of pediatric patients-receiving SCIG, compared with IVIG (∆: – €396.73). Hospital administration (∆: – €2688.03) and IG costs as a function of dosage (∆: – €1927.47) were the main factors affecting the difference in direct costs (Online Resource 5). These factors were offset somewhat by the costs of training for home administration (∆: €694.90) and dispensing (∆: €58.27), which did not apply to IVIG (Online Resource 5). When stratified by age groups, total annual costs for SCIG were lower for both pediatric (∆: – €2521.96) and adult (∆: – €1744.21) patients compared with those for IVIG (Table 3).

**Table 2:** Base-case analysis for PID: total annual cost and time consumed per average patient for IVIG and SCIG

	SCIG	IVIG	Difference
Total cost (€)	14,465.63	18,731.81	− 4266.17
Direct healthcare costs	14,390.90	18,260.35	− 3869.45
Indirect costs	74.73	471.45	− 396.73
Total time (h)	79.24	101.10	− 21.86
Work time	5.32	33.58	− 28.26
School time	4.98	31.70	− 26.72
Leisure time	68.94	35.82	33.12

*IVIG* intravenous immunoglobulin, *PID* primary immunodeficiency diseases, *SCIG* subcutaneous immunoglobulin

**Table 3:** Base-case analysis for PID by age group: total annual cost and time consumed per average patient for IVIG and SCIG

	SCIG		IVIG		Difference	
	Adults (≥ 19 years)	Pediatric (≤ 18 years)	Adults (≥ 19 years)	Pediatric (≤ 18 years)	Adults (≥ 19 years)	Pediatric (≤ 18 years)
Total cost (€)	9014.31	5451.32	10,758.52	7973.29	− 1744.21	− 2521.96
Direct healthcare costs	8984.15	5406.76	10,570.76	7689.59	− 1586.62	−2282.83
Indirect costs	30.16	44.57	187.76	283.70	− 157.60	− 239.13
Total time (h)	30.83	48.41	37.70	63.40	− 6.87	− 14.99
Work time	2.15	3.17	13.37	20.21	− 11.22	− 17.03
School time	0.00	4.98	0.00	31.70	0.00	− 26.72
Leisure time	28.68	40.26	24.33	11.49	4.36	28.76

*IVIG* intravenous immunoglobulin, *PID* primary immunodeficiency diseases, *SCIG* subcutaneous immunoglobulin

### Scenario analyses

The scenario analyses (Table 4) were generally consistent with the base-case analysis. In scenario 1, which accounted for SEFH guidelines to round doses down to the nearest vial in adults and up in pediatric patients, the annual cost of SCIG per average patient (€14,745.11) was 21.8% less than that of IVIG (€18,853.04; ∆: – €4107.93). In scenario 2, which assumed that, in real clinical practice, doses would be rounded up for both adult and pediatric patients, the annual cost of SCIG per average patient (€14,987.42) was 21.5% less than that of IVIG (€19,095.36; ∆: – €4,107.95). In both scenarios, the estimated work, school, and leisure time lost as a result of treatment administration and associated travel and indirect costs were the same as for the base case.

**Table 4:** Scenario analyses: total annual cost and time consumed per average patient for IVIG and SCIG

	Scenario 1^a^			Scenario 2^b^		
	SCIG	IVIG	Difference	SCIG	IVIG	Difference
Total cost (€)	14,745.11	18,853.04	− 4107.93	14,987.42	19,095.36	− 4107.95
Direct healthcare costs	14,670.38	18,381.59	− 3711.21	14,912.69	18,623.91	− 3711.22
Indirect costs	74.73	471.45	− 396.73	74.73	471.45	− 396.73
Total time (h)	79.24	101.10	− 21.86	79.24	101.10	− 21.86
Work time	5.32	33.58	− 28.26	5.32	33.58	− 28.26
School time	4.98	31.70	− 26.72	4.98	31.70	− 26.72
Leisure time	68.94	35.82	33.12	68.94	35.82	33.12

*€* euro, *h* hour, *IVIG* intravenous immunoglobulin, *SCIG* subcutaneous immunoglobulin

^a^Scenario 1: vials adjusted down to the nearest lower dosage in adult patients and the nearest higher dosage in pediatric patients

^b^Scenario 2: vials adjusted up to the nearest higher dosage in both adult and pediatric patients

## Discussion

In this cost-minimization model of IGRT therapies for PID in Spain, the annual cost of SCIG treatment per average patient was approximately one-fifth less than that of IVIG. This difference was largely driven by lower annual hospital administration costs and IG cost as function of dosage associated with SCIG, but was slightly offset by training costs, which did not apply to IVIG. It should be noted that differences in IG costs were mainly due to differences in SCIG and IVIG dosages rather than IG unit costs. Results of both dose-adjustment scenario analyses (i.e., implementing resource-sparing dose adjustments for adults or rounding doses up to the nearest vial for both adults and children) supported the finding from the base case that SCIG was less expensive than IVIG.

A consistent theme has emerged in literature that SCIG administered at home is less expensive than hospital-based IVIG. Analyses using real-world cost data from France, Switzerland, Japan, and Canada have all estimated lower costs of SCIG versus IVIG [30–33]. In a French study using a real-world study sample (*N* = 36), SCIG was 25% less expensive than IVIG because administered SCIG doses were lower than anticipated [30]. A cost-minimization analysis from the Swiss healthcare system perspective found that a total savings of €8,897 per patient could be achieved over 3 years by switching from IVIG administered monthly in an outpatient setting to SCIG administered weekly in the patient’s home [33]. In a Japanese study, patients who switched from IVIG to SCIG had 60% lower productivity loss, resulting in a cost reduction of 10,875 Japanese yen per patient per month [32]. This is generally consistent with the finding in the present analysis that patients-receiving SCIG would lose fewer work hours per year because the treatment is self-administered at home; hence, indirect costs would be lower than for patients-receiving IVIG. A Canadian study found that over 1 year, average hospital costs were lower for home-based SCIG ($1,836) than for hospital-based IVIG ($4,187), and average physician visit costs were lower for SCIG ($84) than for IVIG ($744) [31]. Our analysis further supports the lower direct and indirect costs of home-based SCIG compared with hospital-based IVIG in the setting of the SNS.

Our analysis estimates higher indirect costs for pediatric patients than for adult patients. This is based on author consensus that pediatric patients will generally have parents or caregivers who are not retired, while a proportion of adult patients will be over 65-years old and retired. Therefore, the loss of work activity measured using indirect costs was assumed to be higher for pediatric patients compared to adult patients. A systematic review and meta-analysis of literature on HRQoL in children and adults with PID highlights the need for developing PID-specific instruments to better evaluate the burden of this disease [34].

In contrast to the real-world analyses of SCIG versus IVIG costs, the present cost-minimization analysis did not use real-world administered dosages to model costs. However, to approximate real-world usage, we assumed, in scenario 2, that dosages were likely to be rounded up to the nearest vial in all patients. Furthermore, the present analysis models the combined costs of conventional and facilitated SCIG, which are dosed at different frequencies. The French, Swiss, and Japanese studies assessed the costs of conventional SCIG only, which typically is dosed weekly [30, 32, 33]. We assumed that, during maintenance therapy, conventional SCIG would be administered every 1 to 2 weeks and facilitated SCIG would be administered every 4 weeks. If the difference in dosing frequency between conventional and facilitated SCIG had been considered in the present analysis, the differences in costs would likely have been greater for facilitated SCIG due to the lower frequency of infusions.

The economic crisis of 2008–2009 and subsequent legislative reforms enacted in 2012 have changed the landscape of healthcare spending in Spain. Public spending on healthcare decreased by 12.2% between 2009 and 2015 [35]. Furthermore, average per capita medical expenditures on drugs and medical appliances increased from €365 in 2006 to €427 in 2015, most likely owing to pharmacy cost-sharing reforms [35]. In light of the limited resources available, home-based administration of SCIG in patients with PID warrants attention for its potential to reduce healthcare costs. In the present study, we showed that switching from hospital-based IVIG to home-based SCIG in Spain is potentially cost-saving, consistent with findings in other countries [30–33].

This study had certain strengths and limitations. Although the analysis did not use cost data from claims or other real-world sources, the model inputs and assumptions were based on the real-world experiences of experts and referenced international guidelines. Sensitivity analyses were limited to IG usage scenarios based on clinical guidelines. Only IG products that are approved and reimbursed by the SNS were included. The use of other IG products is marginal and would have had minimal impact on the results. Finally, this analysis was based on Spanish national prices and may not reflect regional differences in costs.

## Conclusions

The findings of this cost-minimization analysis suggest that SCIG is a cost-saving alternative to IVIG for PID in the Spanish healthcare setting; the main factors driving the difference in costs were hospital administration and IG cost as a function of dosage. Patients who receive SCIG can expect to spend fewer hours per year administering IG treatments and traveling to the hospital; consequently, these patients lose fewer hours of work and school than do those who receive IVIG. Together, with patient clinical characteristics, tolerability, preferences, and values, healthcare providers and patients can consider the economic impact of SCIG and IVIG when making treatment decisions.

## Supplementary Information

Below is the link to the electronic supplementary material.

Supplementary file1 (DOCX 17 KB)

Supplementary file2 (DOCX 14 KB)

Supplementary file3 (DOCX 14 KB)

Supplementary file4 (DOCX 14 KB)

Supplementary file5 (DOCX 16 KB)

## Data Availability

Not applicable.
