# Supplementary material for: Cost-minimization analysis of immunoglobulin treatment of primary immunodeficiency diseases in Spain
Source: Eur J Health Econ. 2021 Sep 21;23(3):551–8. doi: 10.1007/s10198-021-01378-x (PMC8964571; doi:10.1007/s10198-021-01378-x)
Supplement: Supplementary file 1 — Supplementary file1 (DOCX 17 KB) [file 10198_2021_1378_MOESM1_ESM.docx]

**Online resource 1**. Usage ratios of SCIG and IVIG per current expert clinical practice

| Immunoglobulin | Usage Ratio (%) |
| --- | --- |
| Facilitated SCIG 10% [1] | 19.1 |
| Conventional SCIG 20% [2] | 10.0 |
| **Total, SCIG** | **29.1** |
| IVIG 5% [3]{, #115;, #115;, #116} | 39.1 |
| IVIG 5% [4] | 2.7 |
| IVIG 10% [5] | 4.5 |
| IVIG 10% [6] | 24.5 |
| **Total, IVIG** | **71.8** |

IVIG, intravenous immunoglobulin; SCIG, subcutaneous immunoglobulin.

References:

1. Spanish Agency for Medicines and Healthcare Products. HyQvia, INN human normal immunoglobulin (SCIg). Available from: https://cima.aemps.es/cima/pdfs/es/p/113840001/P_113840001.html.pdf. Accessed 1 Aug 2019.
2. Spanish Agency for Medicines and Healthcare Products. Hizentra, INN-human normal immunoglobulin. Available from: https://cima.aemps.es/cima/dochtml/p/11687011/P_11687011.html. Accessed 13 Sept 2021.
3. Spanish Agency for Medicines and Healthcare Products. Flebogamma DIF, INN-Human normal immunoglobulin. Available from: https://cima.aemps.es/cima/pdfs/p/07404001/P_07404001.pdf. Accessed 1 Aug 2019.
4. Spanish Agency for Medicines and Healthcare Products. Intratect 50 g/L solution for infusion. https://cima.aemps.es/cima/pdfs/es/p/72294/P_72294.html.pdf. Accessed 1 Aug 2019.
5. Spanish Agency for Medicines and Healthcare Products. Octagamocta 100 mg/mL, solution for infusion. Available from: https://cima.aemps.es/cima/pdfs/es/p/71501/P_71501.html.pdf. Accessed 1 Aug 2019.
6. Spanish Agency for Medicines and Healthcare Products. Privigen, INN-normal human immunoglobulin. Available from: https://cima.aemps.es/cima/pdfs/p/08446001/P_08446001.pdf. Accessed 1 Aug 2019.
