# Supplementary material for: Cost-minimization analysis of immunoglobulin treatment of primary immunodeficiency diseases in Spain
Source: Eur J Health Econ. 2021 Sep 21;23(3):551–8. doi: 10.1007/s10198-021-01378-x (PMC8964571; doi:10.1007/s10198-021-01378-x)
Supplement: Supplementary file 2 — Supplementary file2 (DOCX 14 KB) [file 10198_2021_1378_MOESM2_ESM.docx]

**Supplemental table S2.** Age distribution of patients with PID

| Age Group | Percentage |
| --- | --- |
| Adult population ≥ 19 years | **52.5** |
| Population 19-64 years | 79.5 |
| Population ≥ 65 years | 20.5^a^ |
| Pediatric population ≤ 18 years | **47.5** |
| Population < 5 years | 6.6 |
| Population 5-9 years | 29.3 |
| Population 10-15 years | 43.1 |
| Population 16-18 years | 21.0^b^ |

^a^Data supplied by ESID are based on a population over 59 years old.
^b^Data supplied by ESID are based on a population aged 16-19 years.

ESID, European Society for Immunodeficiencies; PID, primary immunodeficiency diseases.
