# Supplementary material for: Cost-minimization analysis of immunoglobulin treatment of primary immunodeficiency diseases in Spain
Source: Eur J Health Econ. 2021 Sep 21;23(3):551–8. doi: 10.1007/s10198-021-01378-x (PMC8964571; doi:10.1007/s10198-021-01378-x)
Supplement: Supplementary file 3 — Supplementary file3 (DOCX 14 KB) [file 10198_2021_1378_MOESM3_ESM.docx]

**Supplemental table S3.** Dosage of premedication administered with IVIG

| Drug | Population | Route of Administration (% of Patients) | Dose |
| --- | --- | --- | --- |
| Acetaminophen | Adult | Intravenous (50) | 1000 mg |
|  |  | Oral (50) | 1000 mg |
|  | Pediatric | Intravenous (100) | 15 mg/kg |
| Corticosteroid | Adult | Intravenous (100) | 100 mg |
|  | Pediatric | Intravenous (100) | 100 mg |
| Antihistamine | Adult | Intravenous (100) | 6 mg |
|  | Pediatric | Intravenous (100) | 0.15 mg/kg |

IVIG, intravenous immunoglobulin.
