# Supplementary material for: Cost-minimization analysis of immunoglobulin treatment of primary immunodeficiency diseases in Spain
Source: Eur J Health Econ. 2021 Sep 21;23(3):551–8. doi: 10.1007/s10198-021-01378-x (PMC8964571; doi:10.1007/s10198-021-01378-x)
Supplement: Supplementary file 4 — Supplementary file4 (DOCX 14 KB) [file 10198_2021_1378_MOESM4_ESM.docx]

**Supplemental Table S4.** Time consumed for immunoglobulin treatments

| Resource | Facilitated SCIG | Conventional SCIG | IVIG |
| --- | --- | --- | --- |
| Adult preparation and infusion (h) | 1.75 | 1.25 | 3.50 |
| Pediatric preparation and infusion (h) | 1.0 | 1.25 | 3.50 |
| Administration of premedication (h) | - | - | 0.5 |
| Travel to hospital (h) | 0.75^a^ | 0.75^a^ | 0.75 |
| Dispensing of drugs (h) | 0.50 | 0.50 | - |

^a^Only for SCIG infusions that are administered in the day hospital.

h, hour; IVIG, intravenous immunoglobulin; SCIG, subcutaneous immunoglobulin.
