# Supplementary material for: Cost-minimization analysis of immunoglobulin treatment of primary immunodeficiency diseases in Spain
Source: Eur J Health Econ. 2021 Sep 21;23(3):551–8. doi: 10.1007/s10198-021-01378-x (PMC8964571; doi:10.1007/s10198-021-01378-x)
Supplement: Supplementary file 5 — Supplementary file5 (DOCX 16 KB) [file 10198_2021_1378_MOESM5_ESM.docx]

**Supplemental Table S5**. Base-case analysis for PID: Total annual cost and time consumed per average patient for IVIG and SCIG

|  | SCIG | IVIG | Difference |
| --- | --- | --- | --- |
| Direct healthcare costs (€) |  |  |  |
| Immunoglobulin | 13,531.39 | 15,458.86 | -1,927.47 |
| Premedication | 0.00 | 7.12 | -7.12 |
| Hospital administration | 106.34 | 2,794.37 | -2,688.03 |
| Training for home-based SCIG^a^ | 694.90 | 0.00 | 694.90 |
| Dispensing | 58.27 | 0.00 | 58.27 |
| Time consumed (h) |  |  |  |
| Work time |  |  |  |
| Infusion | 2.43 | 23.65 | -21.22 |
| Travel | 2.89 | 9.92 | -7.04 |
| School time |  |  |  |
| Infusion | 2.17 | 22.33 | -20.16 |
| Travel | 2.81 | 9.37 | -6.56 |
| Leisure time |  |  |  |
| Infusion | 58.11 | 25.23 | 32.88 |
| Travel | 3.02 | 10.59 | -7.57 |
| Dispensing | 7.81 | 0.00 | 7.81 |

^a^Training is assumed to be 3-4 sessions of 2 hours each.
h, hour; IVIG, intravenous immunoglobulin; PID, primary immunodeficiency diseases; SCIG subcutaneous immunoglobulin.
